# Vanishing twin syndrome is associated with first-trimester intrauterine hematoma in twin pregnancies after *in vitro* fertilization

**DOI:** 10.3389/fendo.2022.1062303

**Published:** 2023-01-13

**Authors:** Yimeng Ge, Shaoyang Lai, Xiaoxue Li, Jing Shi, Caihong Ma, Jie Zhao

**Affiliations:** ^1^ Center for Reproductive Medicine, Department of Obstetrics and Gynecology, Peking University Third Hospital, Beijing, China; ^2^ Peking University Health Science Center, Beijing, China; ^3^ Department of Obstetrics, Women and Children’s Hospital, School of Medicine, Xiamen University, Xiamen, China; ^4^ National Clinical Research Center for Obstetrics and Gynecology, Peking University Third Hospital, Beijing, China; ^5^ Key Laboratory of Assisted Reproduction (Peking University), Ministry of Education, Beijing, China; ^6^ Beijing Key Laboratory of Reproductive Endocrinology and Assisted Reproductive Technology, Peking University Third Hospital, Beijing, China; ^7^ Department of Pharmacy, Peking University Third Hospital, Beijing, China

**Keywords:** intrauterine hematoma, twin pregnancy, vanishing twin syndrome, fetal cardiac activities, threatened abortion

## Abstract

**Research question:**

Is there an association between intrauterine hematoma (IUH), vanishing twin syndrome (VTS), and subsequent complications in twin pregnancies after *in vitro* fertilization (IVF)? What are the risk factors for these complications?

**Design:**

Women who presented with two live gestational sacs following double embryo transfer were included. Patients with systematic diseases, artificial fetal reduction, and incomplete data were excluded. Further stratification of IUH pregnancies was performed according to IUH-related characteristics (i.e., volume, changing pattern, and relationship with fetal cardiac activities). The primary outcome was the incidence of VTS, while adverse outcomes in the surviving singleton and the gestational age of VTS were secondary outcomes.

**Results:**

The incidence of IUH was 13.8%. A total of 1,078 twin pregnancies including 539 IUH pregnancies and 539 non-IUH pregnancies were included. IUH pregnancy was associated with higher risks of VTS (26.9% *vs.* 18.7%, *p* = 0.001) as well as a higher incidence of preterm birth (*p* = 0.001, crude OR = 1.98, 95% CI 1.28–3.09, adjusted OR = 1.19, 95% CI 1.09–1.24), threatened abortion (*p* < 0.001, crude OR = 9.12, 95% CI 2.90–28.69, adjusted OR = 6.63, 95% CI 1.69–14.67), and postpartum hemorrhage (*p* = 0.024, crude OR = 3.13, 95% CI 1.09–8.99, adjusted OR = 1.16, 95% CI 1.08–1.32) in the surviving singleton. There was no significant difference in risks of other complications. The absence of fetal cardiac activities at the diagnosis of IUH predicted VTS (*p* < 0.001, crude OR 4.67, 95% CI 3.67–5.78, adjusted OR 3.33, 95% CI 1.56–5.14) and fetal loss at smaller gestational age (7.81 ± 2.10 *vs.* 11.39 ± 5.60 weeks, *p* < 0.001), while an IUH with an increasing volume did not increase the risk of VTS but might induce threatened abortion in the surviving fetus (*p* < 0.001, crude OR 1.84, 95% CI 1.32–2.55, adjusted OR 1.72, 95% CI 1.13–2.13).

**Conclusions:**

IUH was a risk factor for VTS in twin pregnancies following double embryo transfer and elevated the risks of threatened abortion, preterm birth, and postpartum hemorrhage in the surviving singleton. The absence of fetal cardiac activities at the diagnosis of IUH elevated the risks of VTS, while an IUH with an increasing volume was associated with threatened abortion without elevating the risks of VTS. An IUH diagnosed before the presence of fetal cardiac activities also resulted in an earlier miscarriage. The study suggests that attention be paid to twin pregnancies with first-trimester IUH to prevent VTS and subsequent adverse perinatal outcomes.

**Highlights:**

First-trimester intrauterine hematoma (IUH) following double embryo transfer is associated with a higher incidence of vanishing twin syndrome (VTS) and elevated subsequent risk of threatened abortion, preterm birth, and postpartum hemorrhage in the surviving singleton. Other perinatal outcomes were not associated with the diagnosis of first-trimester IUH. The absence of fetal cardiac activities at the diagnosis of IUH was of predictive value toward VTS, while an IUH with an increasing size was associated with threatened abortion without elevating the risk of VTS. Incomplete fetal cardiac activities and earlier detection of an IUH might also predict miscarriage at smaller gestational age.

## Introduction

Intrauterine hematoma (IUH) is a common gestational complication characterized by a hypoechoic or anechoic crescent-shaped area found through ultrasonic examinations. The incidence rate of IUH varies from 0.46% to 39.5% (1–3), as different definitions of IUH, inclusion criteria, and ultrasound equipment were applied in diverse study populations (4). Symptoms associated with intrauterine hematoma included vaginal bleeding and pelvic pain, while some cases could be independent of the patient’s subjective symptoms (2, 5). In previous studies, a positive correlation between the presence of an IUH and an uplifting incidence of singleton miscarriages and other adverse perinatal outcomes or insignificant results were reported. Tuuli et al. suggested that women who experienced first-trimester IUH were at a twofold increased risk of both early and late pregnancy loss (6–8). Other perinatal outcomes including placental abruption, preterm premature rupture of the membrane (PPROM), pre-eclampsia, fetal restriction, and preterm delivery were also shown to be of higher incidence (6, 9, 10). However, discrepant findings were reported in some studies where IUH was not associated with adverse pregnancy outcomes (1, 2, 11). Additional controversies lie in the effect of IUH-related characteristics (i.e., volume, location, gestational age at diagnosis, duration, and the presence of vaginal bleeding), as some of these characteristics were shown to impact pregnancy outcomes, whereas other cohorts found no direct correlations (4, 12). Notably, existing literature was mainly about singleton pregnancies following spontaneous conception, and very little research focused on vanishing twin syndrome (VTS) during twin pregnancies following assisted reproduction, which was the theme of our study.

With the growing prevalence of infertility (13) and extensive application of assisted reproductive technology (ART), the rate of twin pregnancy has been increasing accompanying the use of double embryo transfer, which was being performed as a routine ART method in China (14). In prior studies, several studies reported that the frequency of IUH was 12.1%–22.4% in the *in vitro* fertilization (IVF) group (15), which was significantly higher compared with the frequency of 11%–12.4% in the non-IVF group (7, 16), indicating that assisted conception was an independent risk factor for high IUH rates (16, 17). Vanishing twin syndrome, defined as the spontaneous miscarriage of one fetus in the uterine, was estimated to occur in 36% of twin pregnancies (18). Specifically, the occurrence of VTS might also elevate the risk of several perinatal complications, putting both the mother and the fetus at risk (19).

As both IVF and twin pregnancy were found to be associated with either higher rates of IUH or the occurrence of VTS, few reports featured the potential effect of IUH on twin pregnancies after *in vitro* fertilization–embryo transfer (IVF-ET) and the role of IUH-related characteristics in the development of VTS and subsequent complications. Therefore, the purpose of this study was to investigate whether IUH played an important role in altering the risks of VTS and follow-up adverse pregnancy outcomes in pregnant women after IVF-ET and to examine the effect of different IUH-related characteristics (i.e., volume, gestational age at diagnosis, and presence of cardiac activity) on pregnancy complications.

## Materials and methods

### Participants

This was a retrospective observational cohort study conducted in two reproductive centers, the Peking University Third Hospital and Xiamen University Women and Children’s Hospital. The study was approved by the Ethics Committee of Peking University Third Hospital (reference number: IRB00006761-M2020572) and the Ethics Committee of Xiamen University Women and Children’s Hospital (reference number: KY-2022-015-K01).

We reviewed the clinical records and laboratory records of all patients diagnosed with twin pregnancies at the first ultrasound examination after ART between January 2016 and December 2018. In the two centers, all patients underwent routine examination of serum human chorionic gonadotropin (HCG) at 14 and 21 days after embryo transfer and received trans-vaginal sonogram (TVS) at 5 0/7 to 10 6/7 weeks of gestation to identify early pregnancy, subsequent TVS was routinely performed at 2-week intervals until 10–11 weeks of gestation if the pregnancy continued to progress. Further ultrasound examinations and laboratory tests were performed according to standard obstetrical guidelines after 11–12 weeks of gestation (20). Notably, no patients in our study had extra TVS due to the diagnosis of an IUH or the occurrence of uncomfortable symptoms such as vaginal bleeding.

In our study, an IUH was defined by the crescent-shaped collection of fluid between the chorionic membrane and the myometrium, with the largest diameter being determined as the largest of the three orthogonal measurements obtained from the fluid collection area. Non-IUH was defined as the absence of an IUH during the whole gestation period. Gestational age was calculated based on the time of embryo implantation, while the presence of vanishing twin syndrome was defined as vanished or viable fetal intrauterine demise in one of the twins. Gestational age at miscarriage, however, was determined by ultrasound estimation according to the volume of the demise fetal bud. The diagnosis of intrauterine hematoma, measurement of IUH-related characteristics, and the determination of first-trimester pregnancy complications were completed by experienced ultrasound practitioners.

**Figure 1:**
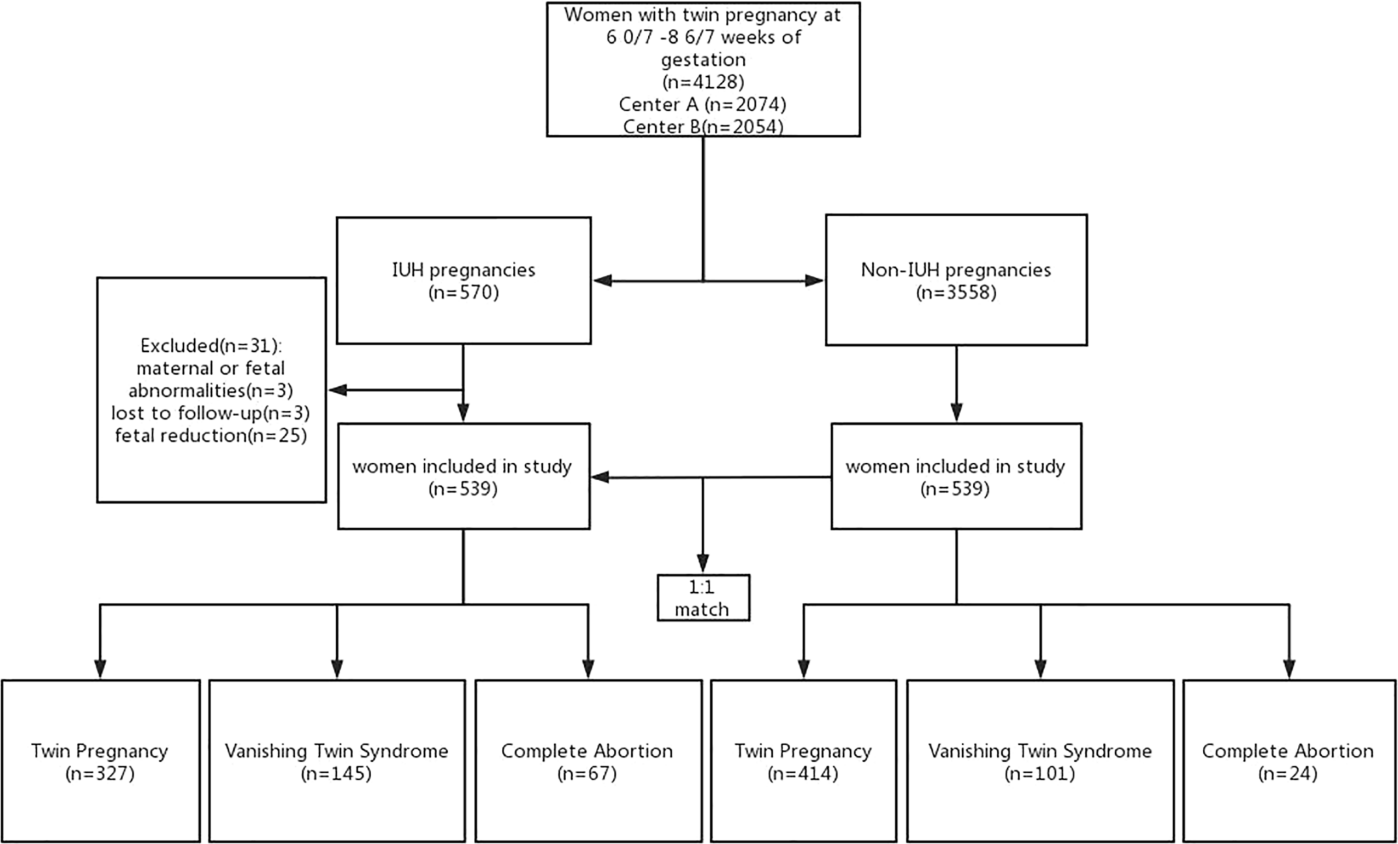
Flowchart showing the study selection of women with or without an IUH pregnancy and the incidence of pregnancy outcomes categorized by the number of live births. Center A, Peking University Third Hospital; Center B, Xiamen University Women and Children’s Hospital; IUH, intrauterine hematoma.

Our inclusion criteria were the presence of two gestational sacs determined by trans-vaginal ultrasound during 5 0/7 to 10 6/7 weeks of gestation. Exclusion criteria included 1) patients with existing maternal high blood pressure, endocrine, and coagulation disorders as well as those with fetal structural malformations and chromosomal abnormalities; 2) patients with uterine factor infertility; 3) fetal reduction; 4) monoamniotic twin pregnancy; 5) lost to follow-up. Therefore, 539 IUH pregnancies were recruited in this study, and 539 non-IUH pregnancies were included after matching with the IUH group in terms of maternal age ( ± 5 years), cycle type, and stage of the embryo during implantation (**Figure 1**).

The IUH group was further classified into subgroups according to the occurring time of an IUH (before or after the presence of fetal cardiac activities) or the changing pattern of the volume of an IUH. The presence of fetal cardiac activities was evaluated *via* an electric database, as all pregnancies were categorized based on whether cardiac activities of the two fetuses were observed at the diagnosis of an IUH. The changing pattern of an IUH was defined by the consecutive ultrasound record, which kept track of the three orthogonal measurements of an IUH and was categorized into those with an increasing volume and those with a decreasing volume. To be exact, we calculated the volume by multiplying three orthogonal measurements of an IUH (measure 1 × measure 2 × measure 3) and multiplying these values by the constant 0.52, as suggested by Campbel (21). Patients who did not obtain at least two ultrasound records were not included in these subgroup analyses, as further classification of IUH pregnancies was performed only in cases with complete data.

### Data collection

Demographic features and clinical information including patients’ medical history, laboratory findings, and ultrasound records were obtained through electrical medical records. Maternal age, body mass index (BMI), cause of infertility, and gestational history were collected as demographic features, while current assisted reproduction records including cycle type (fresh embryo transfer and frozen embryo transfer), stage of the embryo, method of fertilization (*in vitro* fertilization and intracytoplasmic sperm injection), controlled ovarian stimulation (COS) protocols, and pregnancy results were reviewed. Ultrasound records featuring the condition of fetuses and IUH-related characteristics were also documented through the electrical database of the hospital.

### Outcomes measured

Several outcome measures were investigated in this study: the primary outcomes included the rate of VTS, while the secondary outcomes featured the application of cesarean section, the incidence of pre-eclampsia, fetal growth restriction, placental abruption, PROM, postpartum hemorrhage, and threatened abortion, accompanied with neonatal outcomes including low birth weight (<2,500 g), macrosomia (>4,000 g), and preterm birth (<37 weeks). Data related to all outcome measures mentioned above were acquired through either the hospital’s electric database (if the patient delivered in Peking University Third Hospital or Xiamen University Women and Children’s Hospital) or telephone interview (if the patient did not deliver in Peking University Third Hospital or Xiamen University Women and Children’s Hospital) at 24, 28, and 37 weeks of gestation and 42 days after childbirth.

### Statistical analysis

Statistical analysis was performed by using SPSS version 26.0 and R 4.0.4. The continuous variables in accordance with normal distribution were presented with mean ± standard deviations, and an independent t-test was used to compare the results of different studying groups. Those that did not fit the normal distribution were shown as median (25% quartile, 75% quartile) and were compared using the Kruskal–Wallis test. The categorical variables, however, were described as numbers (%) and were compared using the chi-square test. Risk analysis was demonstrated through crude odds ratios (ORs) and 95% confidence intervals (CIs), while adjusted odds ratios were calculated after applying a logistic regression model to adjust for potential cofounders. Correlations between different variables were analyzed using a restricted cubic spline (smooth curve). *p*-Values were considered significant at less than 0.05.

## Results

### Demographic characteristics, elevated risks of VTS, threatened abortion, and postpartum hemorrhage in IUH compared with non-IUH pregnancies

A total of 4,128 pregnant women were diagnosed as twin pregnancy at 6 0/7 to 8 6/7 weeks of gestation after ART cycles were carried out in Peking University Third Hospital Reproductive Center (2,074 women) and Xiamen University Women and Children’s Hospital Reproductive Center (2,054 women) between January 2016 and December 2018. A total of 570 pregnancies were discovered with IUH in the first trimester, and the total incidence rate of IUH was 13.8% (570/4128) in two centers, with 12.1% (250/2074) in Peking University Third Hospital Reproductive Center and 15.6% (320/2055) in Xiamen University Women and Children’s Hospital.

After a retrospective review of all records of 570 twin pregnancies with IUH, 539 women were involved based on the inclusion and exclusion criteria (Center A, n = 238; Center B, n = 301). A total of 539 non-IUH twin pregnancies were included after matching with maternal age, cycle type, and stage of the embryo during implantation of IUH twin women at the proportion of 1:1 (Center A, n = 272; Center B, n = 267) (**Figure 1**).

The demographic characteristics and baseline medical information are shown in **Table 1**, and no differences were observed in patients from different centers except for maternal BMI (**Supplementary Table 1**). In addition, IUH and non-IUH patients received their first ultrasound scan at similar gestational weeks without statistical significance (6.93 ± 0.68 *vs.* 6.88 ± 0.58, *p* = 0.194) and had undergone a similar number of ultrasound scans during the first trimester of pregnancy (2.18 ± 0.56 *vs.* 2.15 ± 0.89, *p* = 0.508). Compared with the non-IUH group, patients who were diagnosed with an IUH had higher rates of VTS. In the IUH group, 12.4% (67/539) patients suffered from complete spontaneous abortion, and 26.9% (145/539) patients had experienced vanishing twin syndrome, while the rate in the non-IUH group was 4.5% (24/539) and 18.7%(101/539) respectively. Other demographic features and pregnancy characteristics were statistically insignificant (**Table 1**).

**Table 1:** Demographic characteristics and clinical information in patients with and without intrauterine hematoma.

Characteristics	IUH	Non-IUH	*p*-Value
	(n = 539)	(n = 539)	
Maternal age (years)	30.86 ± 4.09	30.56 ± 4.02	0.670
Maternal BMI (kg/m²)	22.92 ± 3.25	22.82 ± 3.78	0.778
Duration of infertility (years)	3.72 ± 2.95	3.87 ± 2.86	0.592
Previous gestations			
0	179 (33.2%)	143 (26.5%)	0.053
1	151 (28.0%)	158 (29.3%)	
≥2	209 (38.8%)	238 (44.2%)	
Previous pregnancies			
0	457 (84.8%)	451 (83.7%)	0.877
1	67 (12.5%)	73 (13.6%)	
≥2	15 (2.7%)	15 (2.7%)	
Previous ART cycles			
0	332 (61.6%)	328 (60.9%)	0.803
≥1	207 (38.4%)	211 (39.1%)	
Etiology of infertility			
Female	176 (32.6%)	176 (32.6%)	0.640
Male	190 (35.3%)	191 (35.4%)	
Both	109 (20.2%)	100 (18.6%)	
Idiopathic	64 (11.9%)	72 (13.4%)	
PCOS			
Yes	70 (13.0%)	56 (10.3%)	0.224
No	469 (87.0%)	473 (89.7%)	
Hydrosalpinx			
Yes	149 (27.6%)	130 (24.1%)	0.186
No	390 (72.4%)	409 (75.9%)	
Endometriosis (confirmed by pathology)			
Yes	14 (2.6%)	11 (2.1%)	0.544
No	525 (97.4%)	528 (97.9%)	
Fertilization method			
ICSI	196 (36.4%)	213 (39.5%)	0.286
IVF	343 (63.6%)	326 (60.5%)	
Cycle type			
Fresh	406 (75.3%)	418 (77.6%)	0.389
Frozen	133 (24.7%)	121 (22.4%)	
Stage of embryo			
Cleavage stage	513 (95.2%)	514 (95.3%)	0.886
Blastocyst	26 (4.8%)	25 (4.7%)	
COS protocol			
Antagonist protocol	261 (48.4%)	276 (51.2%)	0.361
Agonist protocol	278 (51.6%)	263 (48.8%)	
Pregnancy outcome			
Complete spontaneous abortion	67 (12.4%)	24 (4.5%)	<0.001***
Vanishing twin syndrome	145 (26.9%)	101 (18.7%)	<0.001***
Surviving twin pregnancy	327 (60.7%)	414 (76.8%)	<0.001***

BMI, body mass index; IUH, intrauterine hematoma; ART, assisted reproductive technology; ICSI, intracytoplasmic sperm injection; IVF, in vitro fertilization; COS, controlled ovarian stimulation; PCOS, polycystic ovary syndrome.

***p < 0.001.

### Comparing other subsequent pregnancy outcomes after VTS between IUH and non-IUH pregnancies

Vanishing twin syndrome was considered to be the most important adverse pregnancy outcome in IUH pregnancies, as the ratio of fetal loss was higher in the IUH group compared with the non-IUH group. To further analyze whether the occurrence of IUH was associated with other adverse pregnancy outcomes in the second or third trimester, we investigated the subsequent perinatal outcomes in patients experiencing first-trimester VTS. Aside from elevated risks of threatened abortion, preterm birth, and postpartum hemorrhage in the surviving singleton, other complications including fetal growth restriction, low birth weight, and placental abruption were not statistically significant between IUH and non-IUH pregnancies (**Table 2**). In general, our data suggested that the occurrence of IUH is associated with the occurrence of threatened abortion, preterm birth, and postpartum hemorrhage followed by the incidence of VTS, while other adverse outcomes were not elevated by first-trimester IUH.

**Table 2:** Incidence and risk of pregnancy complications in VTS patients with and without intrauterine hematoma.

	IUH (n = 145)	Non-IUH (n = 101)	*p*-Value	Crude OR	Adjusted OR^‡^
Preterm birth	57 (39.3%)	20 (19.8%)	0.001**	1.98 (1.28, 3.09)	1.19 (1.09, 1.24)
Cesarean section	91 (62.8%)	58 (57.4%)	0.428	1.09 (0.89, 1.35)	NS
Low birth weight	21 (14.5%)	14 (13.9%)	1.000	1.05 (0.56, 1.96)	NS
Macrosomia	5 (3.4%)	7 (6.9%)	0.240	0.50 (0.16, 1.52)	NS
Pre-eclampsia	5 (3.4%)	3 (3.0%)	1.000	1.16 (0.28, 4.75)	NS
Fetal distress	4 (2.8%)	3 (3.0%)	1.000	0.93 (0.21, 4.06)	NS
Fetal growth restriction	4 (2.8%)	0 (0.0%)	0.146	–	–
Placental abruption	3 (2.1%)	0 (0.0%)	0.271	–	–
PROM	23 (15.9%)	9 (8.9%)	0.126	1.78 (0.86, 3.69)	NS
Postpartum hemorrhage	18 (12.4%)	4 (4.0%)	0.024*	3.13 (1.09, 8.99)	1.16 (1.08, 1.32)
Threatened abortion	39 (26.9%)	3 (3.0%)	<0.001***	9.12 (2.90, 28.69)	6.63 (1.69, 14.67)

IUH, intrauterine hematoma; OR, odds ratio; CI, confidence Intervals; PROM, preterm premature rupture of membranes; NS, not significant.

*p < 0.05; **p < 0.01; ***p < 0.001.

^‡^Adjusted ORs were obtained after matching for age, fertilization method, cycle type, stage of transferred embryos, and previous medical history.

### Correlation between IUH-related characteristics and perinatal outcomes in IUH pregnancies

In order to further investigate how IUH-related characteristics affect VTS and other perinatal outcomes in twin pregnancies, subgroup analyses were performed. First, we tried to analyze whether the occurring time of IUH influenced pregnancy outcomes. All twin pregnancies with an IUH were classified into two groups based on whether IUH was diagnosed before or after the cardiac activity of the fetuses. Among 145 IUH pregnancies with VTS, 142 pregnancies were included while 3 were excluded due to a lack of ultrasound information regarding the discovery of fetal cardiac activities. It was suggested that the presence of an IUH before the discovery of fetal cardiac activities was more likely to result in vanishing twin syndrome (89.5% *vs.* 20.1%, *p* < 0.001, crude OR 4.67, 95% CI 3.67–5.78, adjusted OR 3.33, 95% CI 1.56–5.14) compared with an IUH after fetal cardiac activities. In IUH pregnancies experiencing VTS, a similar analysis was performed to determine the correlation between perinatal outcomes and an IUH diagnosed before or after the establishment of fetal cardiac activities. It turned out that no significant differences were found in other adverse pregnancy outcomes subsequent to VTS in the surviving fetus (**Supplementary Table 2**).

Moreover, in order to analyze how the changing pattern of an IUH affects pregnancy outcomes, we classified IUH pregnancies into two subgroups according to whether the volume of hematoma was increasing or decreasing. A total of 128 pregnancies including 64 pregnancies with an increasing IUH and 64 with a decreasing IUH were included, while 17 were excluded with only one valid documentation of accurate IUH size. We found that the changing pattern of an IUH was not associated with a significant difference in VTS rates, while IUH pregnancies with an increasing volume of hematoma were prone to experience higher possibilities of several adverse pregnancy outcomes than those with a decreasing volume of hematoma. In the surviving singleton, the incidence of threatened abortion was found to be higher (*p* < 0.001, RR 1.84, 95% CI 1.32–2.55) in the former group where the volume of IUH increased during pregnancy. The related data are shown in **Table 3**.

**Table 3:** Incidence and risks of pregnancy complications in IUH pregnancies with an increasing or decreasing volume of hematoma.

	IUH with an increasing volume (n = 64)	IUH with a decreasing volume (n = 64)	*p*-Value	Crude OR	Adjusted OR^‡^
Preterm birth^F^	5 (7.8%)	4 (6.3%)	0.743	1.24 (0.36–4.51)	NS
Cesarean section	37 (57.8%)	43 (67.2%)	0.355	0.86 (0.67–1.14)	NS
Low birth weight	10 (15.6%)	8 (12.5%)	0.800	1.25 (0.53–2.96)	NS
Macrosomia	1 (1.6%)	3 (4.7%)	0.619	0.33 (0.04–3.12)	NS
Pre-eclampsia	2 (3.1%)	1 (1.6%)	1	2.00 (0.19–21.51)	NS
Fetal distress	3 (4.7%)	0 (0.0%)	0.244	–	–
Fetal growth restriction	3 (4.7%)	1 (1.6%)	0.619	3.00 (0.32-28.08)	NS
Placental abruption	3 (4.7%)	0 (0.0%)	0.244	–	–
PROM	12 (18.8%)	9 (14.1%)	0.634	1.33 (0.60–2.94)	NS
Postpartum hemorrhage	6 (9.4%)	7 (10.9%)	1	0.86 (0.31–2.41)	NS
Threatened abortion	47 (74.6%)	26 (40.6%)	<0.001***	1.84 (1.32–2.55)	1.72 (1.13–2.13)
Gestational anemia	13 (20.3%)	11 (17.2%)	0.821	1.18 (0.57–2.44)	

IUH, intrauterine hematoma; OR, odds ratio; CI, confidence interval; PROM, premature rupture of membranes; NS, not significant.

^F^Analysis performed only in cases with complete data.

^‡^Adjusted ORs were obtained after matching for age, fertilization method, cycle type, and stage of transferred embryos.

***p < 0.001.

### The association between gestational age at VTS and IUH-related characteristics

**Figure 2:**
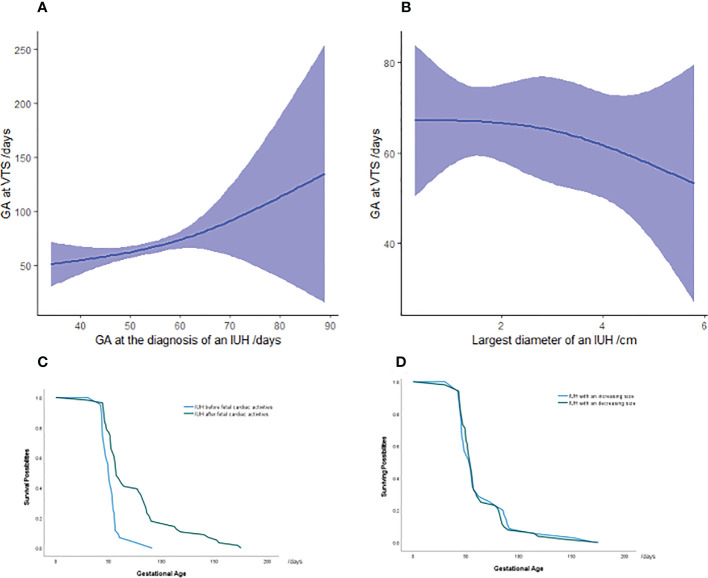
Association between gestational age at miscarriage and IUH characteristics in vanishing twin syndrome patients **(A, B)** Survival analysis in pregnancies with an IUH diagnosis at the presence or absence of fetal cardiac activities **(C)** and an increasing or decreasing volume of hematoma **(D)** in VTS patients. The green and blue lines represent the surviving possibilities of fetuses in vanishing twin syndrome as gestational age progressed. The majority of VTS occurred between 50 and 100 days of gestation, and the y-value represents the percentage of remaining vanishing twins who will be miscarried later. Each color symbolizes IUH patients with the hematoma diagnosed before or after fetal cardiac activity and the either increasing or decreasing volume of an IUH. GA, gestational age; IUH, intrauterine hematoma.

Noticing the higher risks of miscarriage in IUH pregnancies, we applied a non-linear regression model to study the correlation between the gestational age at VTS and IUH-related characteristics including the volume of an IUH, gestational age at IUH diagnosis, presence of fetal cardiac activities at IUH diagnosis, and a changing volume of an IUH. As a result, we found that an IUH diagnosis after the presence of fetal cardiac activities (*p* < 0.001, R^2^ = 0.222) and at later gestational age (*p* < 0.001, R^2^ = 0.262) might be associated with later miscarriage, whereas no additional correlations were found between the gestational age at a fetal loss and other IUH characteristics (**Figure 2**).

## Discussion

This retrospective cohort study indicated that twin pregnancies with IUH after *in vitro* fertilization were primarily associated with higher risks of vanishing twin syndrome. The presence of an IUH was also an essential risk factor for preterm birth, postpartum hemorrhage, and threatened abortion in the surviving singleton during the second or third trimester of pregnancy without affecting the incidence of other adverse perinatal outcomes. Moreover, an IUH diagnosis before the presence of fetal cardiac activities was capable of predicting a higher incidence of VTS and smaller gestational age at miscarriage. An IUH with an increasing size was not associated with a higher incidence of VTS but was at higher risk of threatened abortion.

In this retrospective cohort study, the rate of an IUH in twin pregnancy following ART was approximately 13.8%, which exceeded that of the report by Naqvi et al., where the incidence of IUH in twin pregnancy following both ART and spontaneous conception was 8.9% (22). However, it was lower than the 21.0% incidence rate of IUH in twin pregnancies reported by Ji et al. (21) In the cohort study, we found that IUH in the first trimester was associated with higher risks of vanishing twin syndrome, as well as preterm birth, threatened abortion, and postpartum hemorrhage in the surviving singleton during the second or third trimester. In singleton pregnancy, most of the research suggested that the presence of IUH in the first and second trimesters was associated with miscarriage in singleton pregnancies (6–8). Some existing literature showed that increased risks of fetal loss, pre-eclampsia, placental abruption, and preterm premature rupture of membranes were observed in IUH singleton pregnancies (3, 10, 23).

However, in twin pregnancies after IVF/intracytoplasmic sperm injection (IVF/ICSI), very few studies featured the relevance of IUH in early pregnancy and adverse pregnancy outcomes. Ji suggested that the presence of IUH was associated with the loss of one or both of the fetuses before 20 weeks of gestation, whereas the conception method, IUH size, and previous miscarriage were not independently associated with such fetal loss (21). Different from our study where all twin pregnancies were achieved through IVF/ICSI, Ji’s study population was half conceived naturally and half by an assisted reproductive technique including IVF/ICSI, intrauterine insemination (IUI), and ovulation induction treatment. Another study reported that the finding of a first-trimester subchorionic hematoma (SCH) and the size of the SCH were not associated with adverse pregnancy outcomes in women with twin pregnancies after 24 weeks of gestation. Nonetheless, if the SCH is associated with vaginal bleeding, there is an increased risk of preterm birth. Compared with our study, Mariam’s research did not address the pregnancy outcomes before 24 weeks of gestation, and patients with pregnancy loss prior to 24 weeks of gestation were excluded from the study (22).

In twin pregnancies following the ART method, which comprised approximately 30% of newborns in China, the incidence of fetal loss was reported to be higher than that of singleton pregnancies (24). In previous reports, Zhu et al. suggested a rate of 9.5% vanishing twin in IVF/ICSI twin pregnancy (19) in our center and that VTS was an independent factor for a higher risk of low birth weight (LBW), preterm birth (PTB), small for gestational age (SGA), and perinatal mortality in the surviving singleton. Similarly, more evidence indicated that VTS resulted in poor pregnancy outcomes for the surviving singleton when compared with initial singleton pregnancy in both ART and spontaneous conception (18, 25–27). Additionally, Marton et al. revealed that vanishing twin pregnancies had a lower prevalence and a worse perinatal outcome after IVF-ICSI as compared with those of their spontaneously conceived counterparts (27), and Nigel et al. indicated that the pregnancy outcomes of the surviving singletons that experienced VTS was similar whether by cleavage-stage or blastocyst-stage embryo transfers during fresh IVF cycles (25).

Prior studies did not differentiate the diagnosis of an IUH in the presence or absence of fetal cardiac activities, while in our study, evidence had shown that the absence of fetal cardiac activities yielded predictive value for higher risks of VTS and miscarriage at smaller gestational age since the presence cardiac activities was found to be a symbol of the viability of a fetus in previous reports (28). However, the trend for increased risks of threatened abortion and earlier fetal loss was observed in patients with an increasing volume of IUH. This positive correlation had not been reported in prior studies, as most literature mainly focused on the single detection of an IUH through ultrasound.

The underlying mechanisms for the elevated risks of VTS in IUH pregnancies included secondary mechanical trauma caused by the presence of an IUH, shallow trophoblast invasion, impaired angiogenesis, and resultant friable vessels (29). An IUH might also affect the receptivity of endometrium, which therefore interfered with proper blastocyst–endometrial communication (30) and eventually caused either threatened abortion or VTS. Another explanation might be the fact that the hematoma was caused by the tearing of marginal veins in the placenta (29), which subsequently led to premature perfusion of the intervillous space, causing insufficient development and adaptation of the placenta to cope with oxidative stress and imbalance of free radicals. Such oxidative disorders were followed by the degeneration of syncytiotrophoblast and eventually resulted in VTS, which was the primary yet most essential outcome measure of our study (31). Preterm birth threatened abortion and postpartum hemorrhage could possibly be elevated subsequently, as Zhu et al. reported a trend of more frequent yet severe complications in the surviving singleton following the occurrence of vanishing twin syndrome (19). The presence of threatened abortion in early pregnancy might also bring higher risks of other adverse outcomes such as fetal growth restrictions, pre-eclampsia, PROM, and placental abruption due to the common pathway of underlying placental dysfunction (32).

With regard to the role of fetal cardiac activities, we hypothesized that embryos with cardiac developing activities in a delayed fashion or early formation of an IUH were prone to fail, as multiple organ functions that helped to safeguard the survival of the fetus were less complete in the early stage of embryonic development and were more vulnerable to oxidative stress and the function of mother–fetus interface (33, 34). The enlarging volume of an IUH, however, might associate with higher insufficiency in angiogenesis, a larger scale of endometrium receptivity defects, and heavier mechanical stress of the hematoma (12). The continuing rupture of blood vessels, ongoing detachment of chorionic membrane from the decidua, and the expansion of damage to the definitive villous tissue (30), which promoted the dilation of an IUH, accounted for more severe placental defect and eventually led to VTS and threatened abortion.

In terms of the gestational age at the diagnosis of an IUH and the gestational age at miscarriage, no consensus had been reached in previous studies. Xiang et al. suggested in their 2014 review that most studies featuring the clinical significance of IUH failed to reveal the impact of its concrete characteristics such as size and gestational age at diagnosis; others’ conclusions were limited to the broader application due to the sample size or uncontrolled design (3). In the existing literature, some studies illustrated that an IUH identified in early pregnancy was more likely to result in spontaneous abortion (35), whereas others claimed only those occurring after a certain timeline at gestation yielded deleterious results (36). However, no clear mechanism explaining the timing threshold of the genesis of an IUH and its impact on early miscarriage had yet been reported. We suggested that this correlation might be explained by the idea that earlier development of an IUH probably deteriorated the early development of the placenta or the formation of endometrium receptivity, which further led to the miscarriage of fetuses. Additionally, fetal viability was not formed yet in smaller gestational age and was more vulnerable to the insufficiency of the placenta and the endometrium, resulting in early miscarriage (15, 29, 37).

On the basis of our results, this study provided new knowledge regarding both antenatal and neonatal outcomes of IUH pregnancies following double embryo transfer in assisted conception. Compared with prior studies, our study had the following advantages: a) a large sample volume was applied. b) We analyzed the adverse perinatal outcomes in surviving singleton pregnancy following VTS, establishing the timeline between the occurrence of VTS and pregnancy complications in the surviving fetus during the second or third trimester of pregnancy. c) The cycle types, stage of the embryo, and maternal age were controlled within the electric database, as the role of different cycle types in the onset of IUH had been debated in prior studies. It was reported in some studies that frozen–thawed embryo transfer and blastocyst transfer were risk factors for IUH (24), while Zhou reported that fresh embryo transfer may contribute to IUH onset in IVF/ICSI patients (13). d) Further classifications by the presence or absence of fetal cardiac activities and the changing pattern in the volume of an IUH were performed and analyzed separately in terms of pregnancy outcomes. e) We visualized the correlation between gestational age at miscarriage and different IUH characteristics using a non-linear regression model (smooth curve), as well as the survival possibilities in different groups categorized by those IUH characteristics.

The limitation of this study was the retrospective design. Ultrasound scans during the second or third trimester were not routinely undertaken by ART practitioners, so IUH characteristics subsequent to the first trimester were lacking, making it unable to obtain detailed changing patterns of the volume of an IUH and specific IUH characteristics at the time of delivery (38). Therefore, the major limitation of our study was that IUH characteristics of the second and third trimesters have not been analyzed. Meanwhile, information regarding pharmaceuticals being used during the whole gestational period was not addressed, and the effect of the drug on IUH or adverse pregnancy outcomes might be neglected, as it had been reported that the use of low-dose aspirin might be associated with an increased occurrence and persistence of IUH during the first trimester, regardless of fertility diagnosis or method of fertility treatment (39). In addition, a large number of IUH patients who had undergone artificial fetal reduction were not included in this study. Further prospective studies consisting of more accurate and complete data will be necessary.

To sum up, we speculated that IUH was associated with vanishing twin syndrome and adverse pregnancy complications in the surviving singleton, while the identification of fetal cardiac activities and the measurement of the volume of an IUH presented early warning signs for adverse pregnancy outcomes in both the mother and the fetuses. These findings demonstrated that special attention should be given to twin pregnancies with an IUH in the first trimester to prevent or attend to adverse pregnancy outcomes including VTS, postpartum hemorrhage, and threatened abortion.

## Data availability statement

The raw data supporting the conclusions of this article will be made available by the authors, without undue reservation.

## Ethics statement

The study was reviewed and approved by the Ethics Committee of Peking University Third Hospital (Reference number: IRB00006761-M2020572) and the Ethics Committee of Xiamen University Women and Children’s Hospital (Reference number: KY-2022-015-K01).

## Author contributions

Study design and concept: YG, CM, and JZ. Data collection and analysis: YG, SL, JS, and XL. Interpretation of data and critical revision of the manuscript: YG, CM, and JZ. All authors contributed to the article and approved the submitted version.
