# Supplementary material for: Vanishing twin syndrome is associated with first-trimester intrauterine hematoma in twin pregnancies after in vitro fertilization
Source: Front Endocrinol (Lausanne). 2023 Jan 13;13:1062303. doi: 10.3389/fendo.2022.1062303 (PMC9880446; doi:10.3389/fendo.2022.1062303)
Supplement: Supplementary file 1 [file Table_1.docx]

**Supplementary Table 1 Dempgraphic characteristics and clinical information in patients of two centers**

| Characteristics | Center A | Center B | P-value |
| --- | --- | --- | --- |
|  | (n=510) | (n=568) |  |
| Maternal age(years) | 30.74±3.61 | 30.69±4.43 | 0.838 |
| Maternal BMI(kg/m²) | 22.64±3.66 | 21.41±2.88 | <0.001*** |
| Duration of infertility(years) | 3.67±2.74 | 3.98±3.26 | 0.211 |
| Previous gestations |  |  |  |
| 0 | 149(29.2%) | 173(30.5%) | 0.248 |
| 1 | 114(22.3%) | 195(34.3%) |  |
| ≥2 | 207(40.5%) | 240(42.3%) |  |
| Previous pregnancies |  |  |  |
| 0 | 441(86.5%) | 467(82.2%) | 0.056 |
| 1 | 57(11.2%) | 83(14.6%) |  |
| ≥2 | 12(2.3%) | 18(3.2%) |  |
| Previous ART cycles |  |  |  |
| 0 | 300(58.8%) | 360(63.4%) | 0.125 |
| ≥1 | 210(41.2%) | 208(36.6%) |  |
| Etiology of infertility |  |  |  |
| Female | 153(30.0%) | 199(35.0%) | 0.078 |
| Male | 180(35.3%) | 201(35.4%) |  |
| Both | 106(20.7%) | 103(18.1%) |  |
| Idiopathic | 71(13.9%) | 65(11.5%) |  |
| PCOS |  |  |  |
| Yes | 55(10.8%) | 71(12.5%) | 0.381 |
| No | 455(89.2%) | 497(87.5%) |  |
| Hydrosalpinx |  |  |  |
| Yes | 119(30.0%) | 160(28.2%) | 0.070 |
| No | 391(70.0%) | 408(71.8%) |  |
| Endometriosis(confirmed by surgery) |  |  |  |
| Yes | 9(1.8%) | 16(2.8%) | 0.252 |
| No | 501(98.2%) | 552(97.2%) |  |
| Fertilization method |  |  |  |
| ICSI | 202(39.6%) | 207(36.4%) | 0.285 |
| IVF | 308(60.4%) | 361(63.6%) |  |
| Cycle type |  |  |  |
| Fresh | 398(78.0%) | 426(75.0%) | 0.240 |
| Frozen | 112(22.0%) | 142(25.0%) |  |
| Stage of embryo |  |  |  |
| Cleavage-stage | 490(96.1%) | 537(94.5%) | 0.236 |
| Blastocyst | 20(3.9%) | 31(5.5%) |  |
| COS protocol |  |  |  |
| Antagonist protocol | 250(49.0%) | 287(50.5%) | 0.621 |
| Agonist protocol | 260(51.0%) | 281(49.5%) |  |
| Pregnancy outcome |  |  |  |
| Complete Spontaneous Abortion | 39(7.6%) | 52(9.2%) | 0.076 |
| Vanishing Twin Syndrome | 114(22.4%) | 132(23.2%) |  |
| Surviving Twin Pregnancy | 357(70.0%) | 384(67.6%) |  |

BMI=Body Mass Index, IUH=intrauterine hematoma, ART=assisted reproductive technology, ICSI=intracytoplasmic sperm injection, IVF=in vitro fertilization, COS=Controlled Ovarian Stimulation

*p*<0.001 was marked with***

**Supplementary Table 2 Incidence and risks of pregnancy complications in pregnancies with the diagnosis of an IUH before or after the presence of fetal cardiac activities**

|  | IUH before fetal cardiac activities  (n=68） | IUH after fetal cardiac activities  (n=74) | P-value | Crude OR | Adjusted OR‡ |
| --- | --- | --- | --- | --- | --- |
| Preterm Birth☨ | 4  (5.8%) | 9  (12.2%) | 0.246 | 0.48  (0.15-1.46) | NS |
| Cesarean Section | 44  (64.7%) | 46  (62.2%) | 0.595 | 1.04  (0.84-1.37) | NS |
| Low birth weight | 6  (8.8%) | 14  (18.9%) | 0.096 | 0.47  (0.19-1.15) | NS |
| Macrosomia | 1  (1.5%) | 4  (5.4%) | 0.368 | 0.27  (0.03-2.37) | NS |
| Pre-eclampsia | 1  (1.5%) | 4  (5.4%) | 0.368 | 0.27  (0.03-2.37) | NS |
| Fetal distress | 2  (2.9%) | 2  (2.7%) | 1 | 1.09  (0.16-7.51) | NS |
| Fetal growth restriction | 2  (2.9%) | 2  (2.7%) | 1 | 1.09  (0.16-7.51) | NS |
| Placental abruption | 2  (3.6%) | 1  (1.4%) | 0.607 | 2.18  (0.20-23.46) | NS |
| PROM | 10  (14.7%) | 12  (16.2%) | 0.821 | 0.91(0.42-1.96) | NS |
| Postpartum hemorrhage | 10  (14.7%) | 7  (9.5%) | 0.439 | 1.56  (0.63-3.86) | NS |
| Threatened abortion | 37  (54.4%) | 41  (56.2%) | 0.867 | 0.97  (0.72-1.30) | NS |

IUH=Intrauterine hematoma, PROM=Premature rupture of membrane, OR=Odds Ratio, CI=Confidential Intervals, NS=Not significant

☨Analysis performed only in cases with complete and valid data

‡Adjusted ORs were obtained after matching for age, fertilization method, cycle type and stage of transferred embryos

p<0.05 was marked with*
